# Recent Advances in the Management of Dyslipidemia: A Systematic Review

**DOI:** 10.7759/cureus.81034

**Published:** 2025-03-23

**Authors:** Jacky Xiao Feng Huang, Adil Yousaf, Julie Moon, Ramiz Ahmed, Krishma Uppal, Sudhakar Pemminati

**Affiliations:** 1 Department of Biomedical Education, California Health Sciences University College of Osteopathic Medicine, Clovis, USA

**Keywords:** angiopoietin-like protein 3 inhibitors, antisense oligonucleotides, cholesteryl ester transfer protein inhibitors, dyslipidemia, proprotein convertase subtilisin/kexin type 9 (pcsk9) inhibitors

## Abstract

Dyslipidemia refers to abnormal levels of lipids in the bloodstream, typically exhibiting an increased pattern. Total cholesterol, high-density lipoprotein-cholesterol (HDL-C), low-density lipoprotein-cholesterol (LDL-C), and triglycerides (TGs) are all contributing factors to this disorder. This leads to an increased risk of atherosclerosis and cardiovascular diseases, such as coronary artery disease, which elevates the likelihood of morbidity. Dyslipidemia can be managed via the use of numerous classes of drugs and treatments. The conventional pharmacological agents comprising 3-hydroxy-3-methylglutaryl-coenzyme A (HMG-CoA) reductase inhibitors, selective cholesterol absorption inhibitors, proprotein convertase subtilisin/kexin type 9 inhibitors (PCSK9i), bile acid sequestrants, monoclonal antibodies, and nutritional supplementation, such as inhibitors of cholesterol synthesis and absorption, and promoters of LDL-C excretion, are also discussed. Furthermore, conventional pharmacological treatment of dyslipidemia may elicit a variety of adverse side effects that are detrimental to the quality of life of the user. These side effects include muscle pain, weakness, liver enzyme elevations, and hyperglycemia. This systematic review further analyzes the pharmacological actions of novel lipid-lowering agents such as adenosine triphosphate-citrate lyase inhibitors (ACLi), selective peroxisome proliferator-activated receptor alpha (PPARα) modulators, cholesteryl ester transfer protein inhibitors (CETPi), antisense oligonucleotides (ASO), and angiopoietin-like protein 3 inhibitors (ANGPTL3i) as well as their efficacy in treating dyslipidemia while sparing the user of potentially severe side effects. Compared to existing treatments, novel therapies have shown significantly greater effectiveness in managing dyslipidemia-related lipid profiles and exhibit fewer systemic adverse effects. Some of the recent therapies discussed are alternative treatments that offer patients promising efficacy and improved tolerability. The Preferred Reporting Items for Systematic Reviews and Meta-Analyses (PRISMA) guidelines were followed to ensure a robust and transparent search process, aiming to minimize bias and maximize the retrieval of pertinent studies for review. Thus, this systematic review provides an overview of current and novel treatments for dyslipidemia, describing their efficacy, mechanism of action, safety, and side effects. As experimental investigations and clinical research progress, there is a possibility that a combination of newly tested medications and traditional ones may emerge as a promising treatment option for dyslipidemia in the future.

## Introduction and background

Dyslipidemia is the term for abnormal blood lipid levels, usually shown by decreased levels of high-density lipoprotein (HDL) and raised levels of triglycerides (TGs), cholesterol, and low-density lipoprotein-cholesterol (LDL-C) [1]. This imbalance increases the risk of cardiovascular illnesses such as coronary artery disease and atherosclerosis, which raises morbidity and death rates [2]. Pharmacological interventions such as 3-hydroxy-3-methylglutaryl-coenzyme A (HMG-CoA) inhibitors, selective cholesterol absorption inhibitors, proprotein convertase subtilisin/kexin type 9 inhibitors (PCSK9is), bile acid sequestrants, monoclonal antibodies, and nutritional supplements that promote of LDL-C excretion, and inhibitors of cholesterol synthesis and absorption are used in the traditional treatment of dyslipidemia [3]. While these medications work well to treat dyslipidemia, many patients may experience adverse side effects that impair their quality of life.

Because of these reasons, there has been a rise in interest in studying novel lipid-lowering drugs with unique modes of action that provide better safety profiles in recent years. By examining the mechanisms, effectiveness, and side effects of both standard and non-traditional statin medications, this systematic review aimed to provide a thorough outcome of both established and developing treatments for dyslipidemia.

Pathophysiology of dyslipidemia

**Figure 1:**
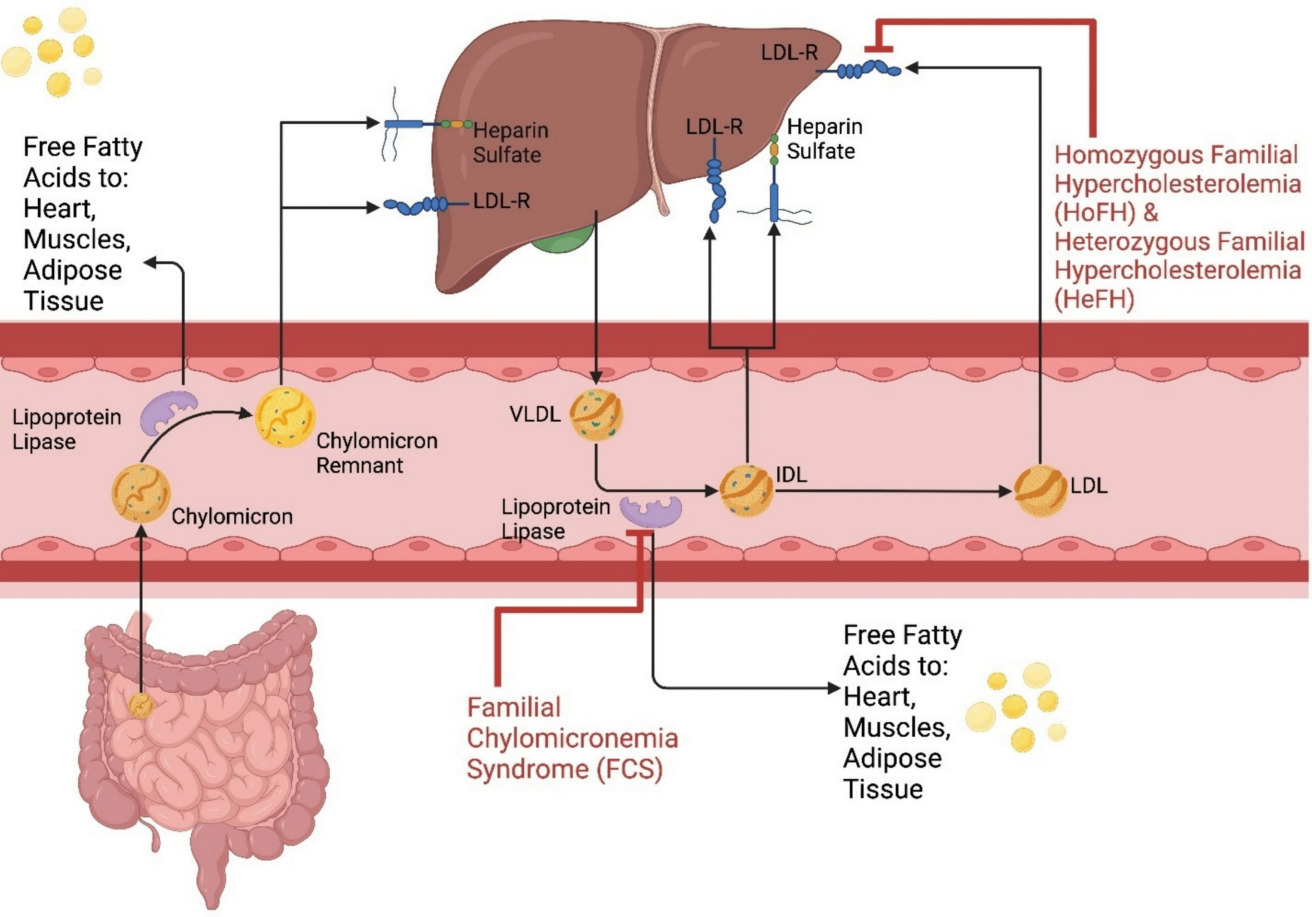
Pathophysiology of dyslipidemia Image credit:  Moon, Julie, Huang, Jacky Xiao Feng, Yousaf, Adil.

Dyslipidemia results from alterations in lipid metabolism caused by an interaction between inherited and environmental factors. Inherited causes of dyslipidemia include familial chylomicronemia syndrome (FCS), homozygous familial hypercholesterolemia (HoFH), and heterozygous familial hypercholesterolemia (HeFH) [4]. Since the liver controls the production, absorption, and excretion of cholesterol and TGs, it is essential for maintaining lipid homeostasis. This equilibrium can be disturbed by dyslipidemia, which leads to high TG and LDL-C levels and low HDL-C (high-density lipoprotein-cholesterol) levels [5]. This imbalance can result in atherosclerosis, a buildup of lipids in arterial walls, raising the risk of cardiovascular events [6]. The process of dyslipidemia can be visualized in Figure 1.

For traditional statins to be successful, they have to inhibit HMG-CoA reductase, which is the enzyme that limits the rate of cholesterol synthesis in the mevalonate pathway. By reducing the quantity of cholesterol generated in the liver and enhancing the clearance of LDL from the bloodstream, this inhibitor aids in achieving a state of lipid balance [7]. However, patients may not always benefit from traditional statins, and they might cause side effects such as hyperglycemia, increased liver enzymes, and muscle pain [8].

Fortunately, some studies show that non-traditional statin drugs that can lower lipid levels through other means are available. Adenosine triphosphate-citrate lyase inhibitor (ACLi) can reduce cholesterol synthesis by inhibiting the ACL enzyme, further inhibiting the HMG-CoA enzyme [9]. PCSK9i blocks the PCSK9 proteins, destroying the liver’s receptors that remove excess cholesterol. As a result, LDL receptor (LDL-R) levels increase, lowering the LDL-C levels in the blood [10]. ANGPTL3i reduces LDL-C levels via a differing mechanism independent of LDL-Rs [11]. ASOs inhibit mRNA synthesis via base-pair hybridization, thereby preventing the production of target proteins involved in LDL-C synthesis [6]. New dyslipidemia therapies beyond traditional statins are emerging, each highlighting their potential to mitigate the side effects of previous treatments [12]. With these new drugs currently being researched, patients with dyslipidemia can be treated with various methods while avoiding the drawbacks of traditional therapeutics and ultimately improving patient adherence and positive outcomes.

The role of dyslipidemia in atherosclerosis and cardiovascular disease

**Figure 2:**
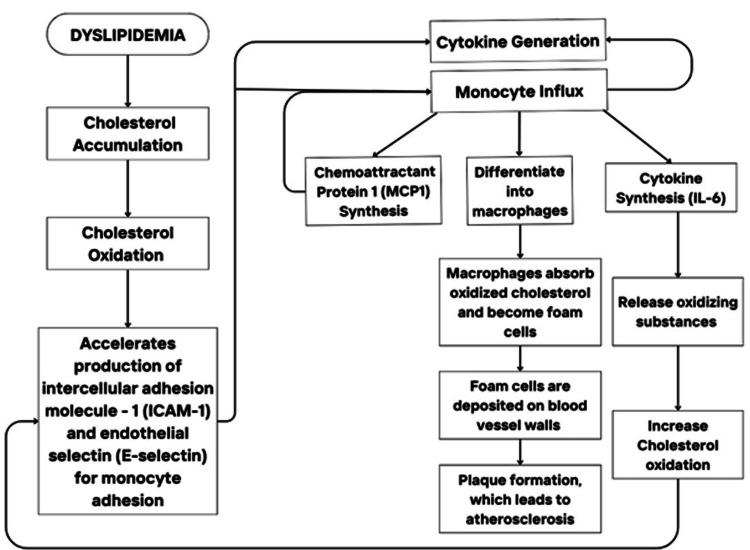
Dyslipidemia’s effects leading to atherosclerosis and cardiovascular disease risk Image credit: Moon, Julie, Huang, Jacky Xiao Feng.

Dyslipidemia, characterized by abnormal blood lipid levels, initiates a cascade of immune-mediated pathological events that lead to the development of atherosclerosis and cardiovascular diseases (CVDs). The production of intercellular adhesion molecule-1 (ICAM-1) and endothelial selectin (E-selectin) by endothelial cells starts when excess cholesterol accumulates within the arterial wall and undergoes oxidation [13,14]. This process attracts monocytes, via chemoattractant protein-1 (MCP-1), that will adhere to the vessel walls. Next, they differentiate into macrophages upon entry into the arterial intima. These macrophages will engulf oxidized cholesterol, forming foam cells which further contribute to plaque formation and fatty streaks on the vessel walls [15,16]. At the same time, interleukin-6 (IL-6) is released, which perpetuates inflammation and oxidation of cholesterol in a positive feedback loop [17-19]. Over time, the deposition of foamy macrophages and immune mediators narrows the blood vessels, leading to increased CVD risk, such as myocardial infarction (MI) and stroke [20,21]. This process can be visualized in Figure 2. The critical interplay of lipid oxidation and immune cell activation in dyslipidemia pathology is an up-and-coming focus of targeted therapies.

## Review

Methods

PubMed/MEDLINE (Medical Retrieval Analysis and Retrieval System Online), Scopus, Web of Science, Elsevier, and Embase databases were accessed to retrieve relevant literature between June and July 2024. We utilized the following keywords: “dyslipidemia,” “dyslipidemia treatments,” “current dyslipidemia treatments,” “novel dyslipidemia treatments,” and “adverse effects of dyslipidemia therapies.” Search terms regarding each specific medication were also utilized. The PRISMA guidelines were followed to efficient search process, aiming to minimize bias and maximize the retrieval of pertinent studies for review [22].

**Figure 3:**
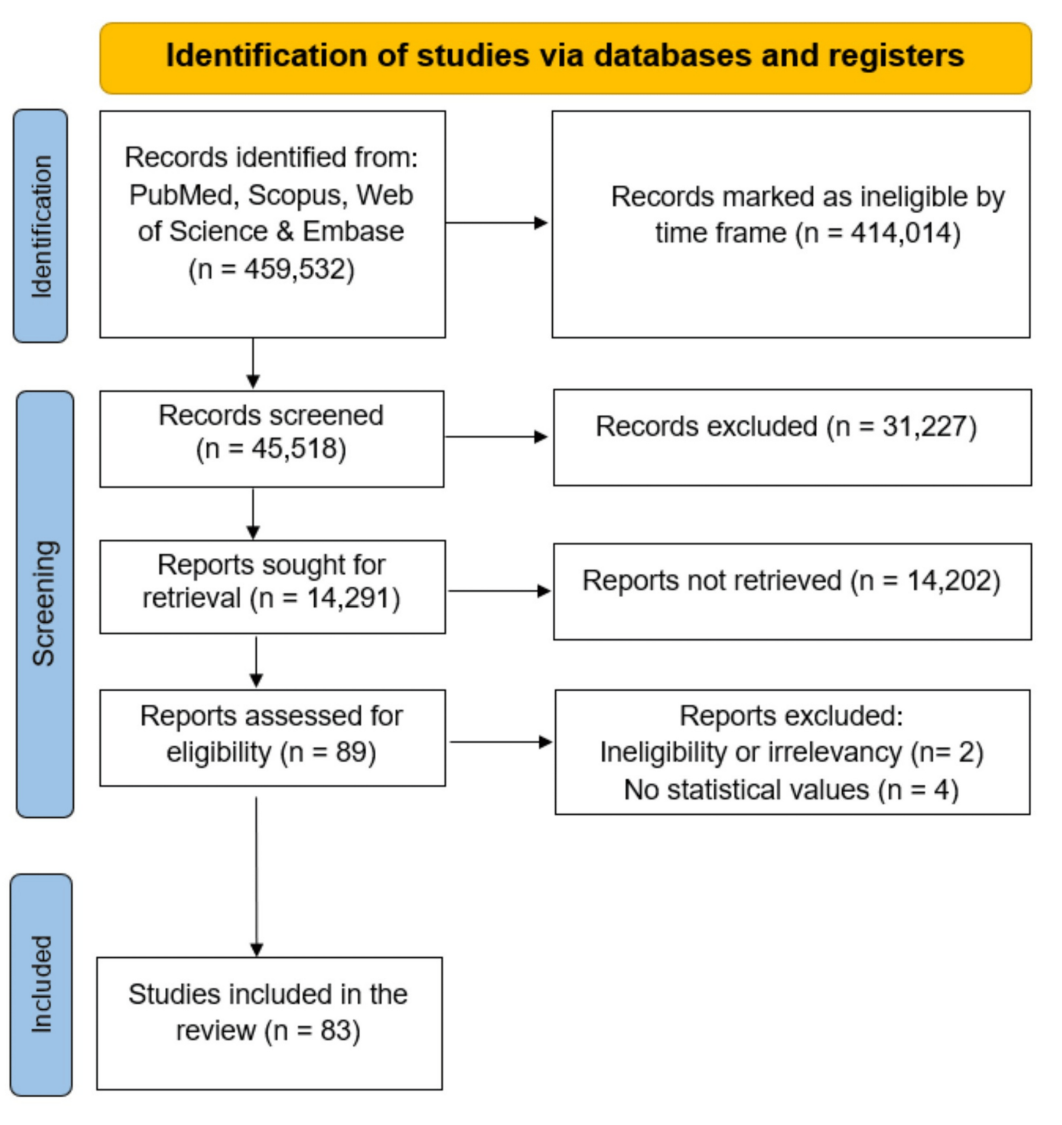
The PRISMA flow diagram depicting the study selection process PRISMA: Preferred Reporting Items for Systematic Reviews and Meta-Analyses.

The search was limited to clinical trials, genetic studies, meta-analyses, and reviews published in peer-reviewed journals for novel and current medications. Journals involving clinical trials were used for developing medications to indicate effectiveness in a smaller population. We extracted data on authors, publication year, study design, sample size, treatment outcomes, and key findings on the adverse effects of both current and novel dyslipidemia therapies. From selected studies, relevant information such as the mechanism of action, benefits, and side effects was reviewed and summarized to provide a comprehensive overview of pharmacological treatments. Additionally, for novel upcoming treatments, only trials from 2014 to 2025 were obtained. Duplicate studies, incomplete studies lacking control groups, and articles that were not in English were excluded. From there, a manual selection for appropriate drugs and drug categories was done to ensure the inclusion of any additional publications or trials that might have been missed by the electronic search and the exclusion of any articles ineligible or irrelevant to the systemic review. Ultimately, 83 studies met our stringent criteria and were included in the review. Studies of specific drugs that express statistical values such as significance, p-values, confidence ratios, and hazard ratios (HRs) were selected to be presented in a tabular format. The study selection process is visualized in Figure 3.

Current treatments 

Statins

Statins are a class of medications that reduce LDL-C by inhibiting the HMG-CoA reductase enzyme. While statins are conventionally the first-choice therapy in managing cholesterol and the risk of cardiovascular events, they present with notable side effects such as statin-associated muscle symptoms (SAMS) [23]. A recent clinical study by Mangone et al. found that 44% of the patients (17 out of 39) experienced a set of muscular symptoms while on statin therapy. SAMS were evaluated and confirmed through a Brief Pain Inventory score, with patients on statin treatment (1.75 ± 0.6) receiving a significantly higher score than those with unconfirmed SAMS (0.66 ± 0.21, p ≤ 0.01). There were no significant changes in tissue oxygenation during handgrip exercise between participants on statin therapy and placebo: -2.4% (95% CI: -6.1 to 1.4, p > 0.05), and no significant changes in creatine kinase levels (p > 0.05) [24]. Genomic studies by Murphy et al. attributed drug-induced SAMS to genetic disposition, with increased risk correlating to transmembrane protein 9 (TMEM9) gene variants, reporting an odds ratio (OR) of 1.39 (95% CI: 1.24-1.55; p = 6.01 × 10⁻⁹). This study showed another gene involving SAMS other than the previously implicated solute carrier organic anion transporter family member 1 B1 (SLCO1B1) gene for atorvastatin and rosuvastatin-induced SAMS [25]. SAMS has been a key reason for patients to discontinue statin therapy. In response, a clinical study by Hlatky et al. examined whether vitamin D supplementation can improve SAMS and reduce statin discontinuation rates in 2,083 new statin users. In this study, over 31% of participants reported muscle symptoms in both the vitamin D and placebo groups; the adjusted OR was 0.97 (95% CI: 0.80-1.18; p = 0.78). There was also no significant difference in the statin discontinuation rate, 13% in both groups, and the adjusted OR was 1.04 (95% CI: 0.80-1.35; p = 0.78). This study demonstrates that vitamin D is insignificant in reducing the incidence of SAMS in statin-therapy patients [26]. Patients on long-lasting statin therapy have also shown an increased risk of developing type 2 diabetes mellitus (T2DM). A recent cohort study by Ko et al. has demonstrated that statin users had a diabetes incidence rate of 13.4 events per 1000 person-years, nearly doubling the non-users' incidence rate at 6.9 events. The adjusted HR for diabetes mellitus in statin users versus non-users was 1.88 (95% CI: 1.85-1.93) [27]. Collectively, these studies highlight the complexity of the adverse effects of statin therapy. These studies emphasize the importance of considering genetic factors, variability in drug-induced symptoms, and limited research on the effectiveness of interventions that mitigate side effects when starting statin therapy. The findings are summarized in Table 1. 

**Table 1:** Role of statins in the management of dyslipidemia SAMS: statin-associated muscle symptoms; LDL-C: low-density lipoprotein-cholesterol; CV: cardiovascular; HMG-CoA: 3-hydroxy-3-methylglutaryl-coenzyme A; TMEM9: transmembrane protein 9; HDL-C: high-density lipoprotein-cholesterol; SLCO1B1: solute carrier organic anion transporter family member 1B1.

Medication, author, year, country	Study population	Mechanism of action	Benefits	Adverse effects
Statin: Mangone et al., 2024, USA [24]	N = 39	HMG-CoA reductase inhibitions reduce LDL-C	Effective lipid-lowering to reduce CVD risk	SAMS: 44% reported muscle symptoms; significant pain inventory scores
Genomic studies: Murphy et al., 2022, USA [25]	N = 11,880	Genetic predisposition of SAMS involving TMEM9 and SLCO1B1	Identification of genetic variants increasing SAMS risk	Significant odds ratio of SAMS with TMEM9 1.39 (95% CI: 1.24–1.55; p = 6.01 × 10⁻⁹)
Statin and Vitamin D: Hlatky et al., 2023, USA [26]	N = 2083	Vitamin D supplementation with statins	No improvements in SAMS incidents or statin discontinuation rates	Muscular symptoms: 31% in both groups, discontinuation rate: 13% in both groups.
Statin: Ko et al., 2019, South Korea [27]	N = 2,162,119	Inhibits HMG-CoA reductase and reduces LDL cholesterol synthesis	CV events prevention in high-risk individuals	Associated with dose-dependent increased diabetes risk. Incidence: 13.4 events/1000 person-years, nearly double the non-users

Ezetimibe

Ezetimibe is a cholesterol absorption inhibitor that has been used for the treatment of dyslipidemia and hypercholesterolemia. It works by inhibiting the Niemann-Pick C1-Like 1 (NPC1L1) protein, disrupting the absorption of cholesterol in the small intestine [10]. This drug is often administered alongside traditional statins, as beneficial results have been recorded. Kim et al. describe the RACING trial, in which 3,780 patients with atherosclerotic cardiovascular disease (ASCVD) were randomly assigned to receive either a moderate-intensity regimen of ezetimibe and rosuvastatin combined or a high-intensity rosuvastatin monotherapy. After three years, LDL-C levels were below 70 mg/dL in 72% of the patients in the combination group, whereas only 58% of patients achieved this goal in the rosuvastatin monotherapy group (p < 0.0001) [28]. Eighty-eight patients in the combination group and 150 patients in the statin monotherapy group either had a dosage reduction or discontinued treatment, highlighting differences in intolerance [28]. Kim et al. concluded that ezetimibe, when combined with a statin, provides a greater reduction in cholesterol levels and reduces the occurrences of adverse effects, such as drug intolerance, from traditional statin monotherapy. Although the effects of ezetimibe utilized as a combination therapy have been studied, more research needs to be done on the long-term effects of ezetimibe as a monotherapy for cholesterol reduction. One such study, however, highlights the positive impact of ezetimibe. In this review, the authors discuss the EWTOPIA 75 trial in which Japanese patients were given 10 mg of ezetimibe to reduce the incidence of CVD. LDL-C levels were significantly reduced by 25.9%, and the risk of CVD was lowered (HR 0.66, 95% CI 0.50-0.86) [29]. The findings are summarized in Table 2.

**Table 2:** Role of ezetimibe in the management of dyslipidemia LDL-C: low-density lipoprotein-cholesterol; NPC1L1: Niemann-Pick C1-Like 1.

Medication, author, year, country	Study population	Mechanism of action	Benefits	Adverse effects
Ezetimibe: Kim et al., 2022, South Korea [28]	N = 3780	Cholesterol absorption inhibitor, blocks Niemann-Pick C1-Like 1 (NPC1L1) protein, preventing cholesterol uptake in the small intestine	LDL-C < 70 mg/dL in 72% of patients (combination) vs. 58% (monotherapy)	Treatment discontinuation due to intolerance: 88 patients (4.8%) in the combination group vs. 150 patients (8.2%) in the monotherapy group

Fibrates

Fibrates are a class of medications used to treat dyslipidemia by activating the transcription factors peroxisome proliferator-activated receptors (PPARs) to reduce the level of TGs, triglyceride-rich lipoprotein cholesterol (TRL-C), and Lp(a) by stimulating fatty acid oxidation and increasing lipoprotein lipase (LPL) synthesis [30].

Fenofibrate

In a double-blind placebo-controlled study by d'Emden et al., the use of fenofibrate monotherapy in naïve-statin individuals showed that both sexes (male and female) had significant reduction of LDL, triacylglycerol (TAG) levels, and total cholesterol. Women overall showed more improvement with 14.0% total cholesterol reduction (p < 0.001) compared to 9.9% in men at four months and 9.5% reduction (p < 0.001) compared to 5.2% in men after the study. Women also showed a greater reduction in LDL (p < 0.001) at 16.5% versus 9.4% in men at four months and 9.8% versus 3.3% after the study. The study also found that fenofibrate was able to decrease the percentage of female patients with dyslipidemia from 42.7% to 23.9% in women (p < 0.001) and male patients from 34.0% to 20.2% in men (p < 0.001) [31]. 

Statins and fenofibrates are often used as combination therapy for the treatment of dyslipidemia, as evidenced in a study by Ezhov et al. The patients in the study were those who were on stable therapy with statins with TG levels above 2.3 mmol/L and were prescribed fenofibrate 0 to 3 days before the study. Ezhov et al. found that at six months (p < 0.001), baseline TG levels were reduced by 50.1% and baseline non-HDL-C levels reduced by 33.7% (both p < 0.001). There was a total cholesterol reduction of 24.7% (p < 0.001) and a low-density lipoprotein (LDL) level reduction of 25.5% (p < 0.001) during the follow-up after the conclusion of the study. There was also a more than 39% reduction of C-reactive protein compared to baseline [32]. Regarding the safety and adverse effects of fenofibrate, the medication is not recommended during pregnancy due to animal studies showing complications such as “delayed delivery, reduced birth weight, increased post-implantation loss, skeletal and visceral abnormalities, abortions, and fetal deaths” [33]. Otherwise, the drug is well tolerated, with Ezhov et al. only reporting one mild adverse drug reaction in a patient taking warfarin.

Gemfibrozil

Recent studies proposed that gemfibrozil can inhibit statin metabolism through the glucuronidation pathway, leading to an increased plasma concentration of statins and their associated side effects, such as SAMS [30]. Moreover, clinical studies by Wiggins et al. confirm a two- to three-fold increased risk of statin toxicity when using gemfibrozil with simvastatin and lovastatin (p < 0.001). Pravastatin coadministration with gemfibrozil increases plasma concentrations of pravastatin by 202% (p < 0.0001). The authors attributed a 15-fold increased risk of adverse effects such as myalgia, myopathy, and rhabdomyolysis from gemfibrozil inhibiting CYP2C8 and OATP1B1 transporters, which leads to increased statin systemic exposure [34]. Another notable adverse effect of gemfibrozil is the disruption of phospholipid and bile acid homeostasis, which leads to hepatotoxicity from chronic activation of PPAR-α. Liu et al.’s preclinical mouse studies found that lysophosphatidylcholine (LPC) levels increased significantly in wild-type mice on day 7 of gemfibrozil (p < 0.05) and increased taurocholic acid by 13-fold in wild-type mice after day 14 of gemfibrozil (p < 0.05). Additionally, these mice showed a 43% liver weight increase compared to PPARα-null mice without gemfibrozil, suggesting drug-induced hepatomegaly. Histopathological analysis revealed fatty liver and elevated ALP levels, suggesting bile duct and hepatocyte toxicity [35]. These studies of adverse effects highlight a few reasons why gemfibrozil has fallen out of favor as a therapy of choice to treat dyslipidemia. The findings are summarized in Table 3.

**Table 3:** Role of fibrates in the management of dyslipidemia PPAR: peroxisome proliferator receptor; LDL: low-density lipoprotein-cholesterol; CVD: cardiovascular disease; TG: triglyceride; TCA: taurocholic acid; CYP2C8: cytochrome P450, family 2, subfamily C, polypeptide 8; OATP1Bi: organic anion transporting polypeptide 1B1; LPC: lysophosphatidylcholine; INR: international normalized ratio.

Medication, author, year, country	Study population	Mechanism of action	Benefits	Adverse effects
Fenofibrate: d'Emden et al., 2014, UK [31]	N = 9,795	PPARs activator	Reduces CVD events in patients with dyslipidemia	Slight risk in women for pancreatitis and pulmonary embolism
Fenofibrate: Ezhov et al., 2023, Russia [32]	N = 988	PPARs activator	Reduces total cholesterol and LDL in patients with dyslipidemia	Complications may occur if taken during pregnancy
Gemfibrozil: Wiggins et al., 2016, USA [34]	N > 1000	Inhibits CYP2C8 and OATP1B1 transporters	Reduces TG by up to 35% in patients with hypertriglyceridemia	Co-administration with statins increases the risk of rhabdomyolysis by up to 15-fold, with statin plasma concentration rising 2-3x
Gemfibrozil: Liu et al., 2014, USA [35]	N =15 wild-type mice and PPARα-null mice	PPARs activator disrupting LPC and TCA homeostasis	Reduces TG and lipid levels but not the focus of study	Hepatomegaly, hepatotoxicity, and fatty liver. 43% increase in liver/body ratio. Bile acid disruption with a 13-fold increase in TCA

Microsomal TG Transfer Protein Inhibitors

Lomitapide: Lomitapide is a microsomal triglyceride transfer protein inhibitor (MTTPi) used to lower blood cholesterol levels in individuals who are affected by dyslipidemia disorders such as FCS and HoFH. It inhibits the creation and dispersion of apolipoprotein B (ApoB)-containing lipoproteins within the intestine and liver by binding to MTTP and preventing lipid transfer [36]. The "LOCHNES" study by Cefalù et al. demonstrated that lomitapide significantly reduced TG and ApoB levels in patients with FCS, finding that the median of individual changes from baseline percentage for TG was -70.5 (-90.7, - 48.0), p-value <0.0001 and that of ApoB was -43.8 (-66.3, -25.2), p-value <0.0001. Moreover, there was an increase in HDL with the median of individual changes from baseline percentage of +20.7 (+33.3, 15.0), p-value <0.01. The most common (55.6%) adverse effect noted in the study was gastrointestinal (GI)-related [36].

Lomitapide can also be administered in combination with evinacumab. In a case study by Tada et al., a young male patient with HoFH showed lowered LDL cholesterol levels with the addition of lomitapide at 17 years old after failure to see sufficient reduction of LDL levels with evolocumab, statins, ezetimibe, and lipoprotein apheresis. A year later, evinacumab was added to his treatment regimen, which helped further reduce LDL levels and allowed for a decrease in the frequency of lipoprotein apheresis. Overall, the significant LDL reduction and carotid plaque regression achieved through the combination of lomitapide with evinacumab led to improvement in quality of life and no significant side effects during long-term follow-ups, but specific p-values were not provided [37]. This study indicates a promising future combination of therapies utilizing lomitapide for the treatment of HoFH. The findings are summarized in Table 4.

**Table 4:** Role of MTTP inhibitors in the management of dyslipidemia FCS: familial chylomicronemia syndrome; LFT: liver function test; GI: gastrointestinal.

Medication, author, year, country	Study population	Mechanism of action	Benefits	Adverse effects
Lomitapide: Cefalù et al., 2022, Italy [36]	N = 18	Microsomal triglyceride transfer protein (MTTP) inhibitor	Reduces TG levels in FCS patients	Mild to moderate GI concerns (40%) and increased liver fat. Elevated LFTs (10-13%)

Diacylglycerol Acyltransferase Inhibitors (DGATis)

Pradigastat: Pradigastat is a DGATi with the potential for treating subtypes of dyslipidemia, such as familial chylomicronemia disorder (FCS). Meyers et al. investigated six FCS members in an open-label clinical trial that lasted seven days. Members were under strict dietary control to set a standard. The trial consisted of three, partitioned 21-day treatment periods in which pradigastat was administered consecutively at doses of 20 mg daily, 40 mg daily, and 10 mg daily. Each treatment period was isolated by a four-week washout period to reduce carryover impacts. After each treatment period, fasting and postprandial TG and apoB48 levels were evaluated [38].

The results illustrated a critical dose-dependent decrease in TG levels. Administration of pradigastat at 20 mg caused a mean diminishment of 41% in TG levels. The 40 mg treatment period accomplished a more prominent diminishment of 70% (p < 0.01 for both). These findings reflect the dose-dependent impacts of pradigastat on diminishing chylomicron TG substance and apoB48 levels [38].

The trial also proved that pradigastat was well tolerated, with only minor GI effects being reported. Overall, these results highlight the potential of pradigastat as a focused treatment for overseeing dyslipidemia in patients with FCS.

Ervogastat: Ervogastat is a diacylglycerol acyltransferase-2 inhibitor (DGAT2i) that blocks the catalyzation of the final step of TG synthesis. By doing so, ervogastat decreases TG levels and mitigates dyslipidemia-related complications without the GI side effects observed in DGAT1i. The majority of ervogastat research shows efficacy in treating NASH and is currently being investigated for its role in lipid metabolism and dyslipidemia management. Løvsletten et al. explored the effects of DGAT1 and DGAT2 inhibitors on lipid metabolism in human skeletal muscle cells of eight healthy donors. In comparison to DGAT1i, DGAT2i showed limited effects on TAG synthesis from exogenous FFAs; however, it showed a significant reduction in TAG synthesis from glycerol by ~60% in the absence of oleic acid (OA) and ~25% with OA (p < 0.05). Furthermore, DGAT2i decreased fatty acid oxidation by reducing CO_2_ production (p < 0.05) [39]. Amin et al. conducted three double-blind, placebo-controlled phase 1 trials where patients received ascending doses of ervogastat from 30 to 600 mg daily over 14 days while assessing fasting TG and GI adverse events often associated with DGAT1i. This study showed a maximum fasting TG reduction of 23.79% (95% CI: -34.01 to -11.99; p = 0.006) at the highest dose (600 mg) and a 22.46% reduction in TG area under the curve (AUC) at 240 mg (95% CI: -32.31 to -11.18; p = 0.008). Patients reported mild fatigue and headache, but it was not statistically significant compared to placebo (p = 0.82) [40]. Ervogastat offers a promising therapeutic approach for dyslipidemia in effectively reducing TG levels. These findings support further investigation and the potential to integrate DGAT2i into the standard care of dyslipidemia, preventing CVD. The findings are summarized in Table 5.

**Table 5:** Role of DGAT inhibitors in the management of dyslipidemia DGAT: diacylglycerol acyltransferase; TG: triglyceride; GI: gastrointestinal.

Medication, author, year, country	Study population	Mechanism of action	Benefits	Adverse effects
Pradigastat: Meyers et al., 2015, USA [38]	N = 6	Inhibits DGAT1, reducing chylomicron triglyceride synthesis and secretion, and lowering postprandial TG and apoB48 levels	Reduction in fasting TG levels: 41% (20 mg dose) and 70% (40 mg dose)	GI events (i.e. diarrhea and abdominal pain)
DGAT2i: Løvsletten et al., 2020, Norway [39]	N = 8	Inhibits DGAT2, reducing de novo TAG synthesis from glycerol-3-phosphate	Reduced TAG synthesis (60% without OA, and 25% with OA)	Reduced fatty acid oxidation
Ervogastat: Amin et al., 2023, USA [40]	N = 125	Inhibits DGAT2, blocking final step of TG synthesis	23.79% reduction in fasting TG at 600 mg and 23.79% at 240 mg	Mild fatigue and headache in ~4% of patients. No significance compared to placebo patients

Bile Acid Sequestrants

Bile acid sequestrants prevent the intestines from reabsorbing bile acids, therefore treating dyslipidemia. They work by attaching to bile acids in the GI tract, thereby creating an insoluble complex that can be removed in the stool [40]. This process reduces serum LDL-C levels by disrupting the enterohepatic circulation of bile acids and allowing the liver to produce additional bile acids from cholesterol. Depending on the agent and dosage, bile acid sequestrants can reduce LDL-C by 7-30%, and they are thought to be effective and safe [41]. Side effects include constipation, stomach pain, bloating, vomiting, heartburn, loss of appetite, indigestion, and upset stomach [41]. In addition, bile acid sequestrants may interfere with the absorption of other medications, such as digoxin, warfarin, and thyroid hormones [41]. Therefore, it is important to administer bile acid sequestrants with attention. For individuals who do not respond well to statins and/or require additional effectiveness, bile acid sequestrants can be used as an adjuvant therapy.

Colesevelam: Colesevelam is a bile acid sequestrant that was Federal Drug and Administration (FDA) approved in 2009. According to a post hoc review of pooled clinical trial data, colesevelam was effective in treating dyslipidemia in a variety of age groups. For both older (≥65 years, n = 154) and younger (<65 years, n = 381) patients with hyperlipidemia, colesevelam resulted in comparable mean decreases in LDL-C from baseline when compared to placebo (-16.6% vs. +0.5% and -13.7% vs. +0.4%, respectively) [42]. Colesevelam demonstrated similar efficacy in older patients (≥65 years, n = 154) and younger patients (<65 years, n = 381) with T2DM, with notable decreases in LDL-C (-14.7% in older and -15.5% in younger patients, both p < 0.001) and A1C (treatment difference of -0.59% in older and -0.54% in younger patients, both p < 0.001) [42]. The medication was usually well tolerated, and the incidence of side effects was comparable across age groups. However, hypoglycemia was somewhat higher in T2DM individuals. In elderly patients, colesevelam was more common than a placebo (5.8% vs. 2.3%), although no major hypoglycemia episodes were reported [42]. Adverse effects include constipation (<65 years age = 10.3%, >65 years age = 13.4%), dyspepsia (8.8%, 6.7%), nausea (4.7%, 2.6%), accidental injury (3.4%, 4.6%), asthenia (4.1%, 2.1%), pharyngitis (3.6%, 1.0%), flu syndrome (3.4%, 2.1%), rhinitis (3.1%,3.6%), and myalgia (2.6%, 0.5%) [42]. According to these findings, colesevelam is equally safe, well-tolerated, and successful in treating glycemic control and dyslipidemia in both younger and older adult populations.

Colestipol: Colestipol is another bile acid sequestrant that was FDA approved in 1977. In an article discussing non-statin therapies, a previous study analyzing colestipol for hypercholesterolemia was explored. Participants, who had 250 mg/dL of total cholesterol or more for up to three years, were either treated with colestipol or a placebo. After one month of the study, colestipol showcased a significant decrease in total cholesterol by 32 mg/dL (p < 0.001) and an increase in TGs by 33 mg/dL (p ≤ 0.02) compared to the placebo [43]. Men experienced a significant reduction in cardiovascular-related deaths (p ≤ 0.02), while women did not [43]. Adverse effects include GI issues [43]. Additionally, colestipol is often explored in other studies when combined with niacin [43]. All in all, colestipol is a valuable bile acid sequestrant in managing cholesterol levels.

Cholestyramine: Cholestyramine, a bile acid sequestrant, was FDA approved in 1973. A study with nine healthy volunteers (four men and five women) participated in the study, which showed that cholestyramine was an effective treatment for dyslipidemia. Cholestyramine dramatically lowered total serum cholesterol levels in the long-term treatment experiment, and the effect lasted for six days following treatment discontinuation (p < 0.05) [44]. Additionally, as seen by increased lathosterol/cholesterol ratios (p < 0.001), the medication caused a substantial increase in cholesterol production [44]. Notably, in both acute and long-term studies, cholestyramine caused circulating fibroblast growth factor 19 (FGF-19) levels to drop by >90%, which in turn caused bile acid production to rise (p < 0.001) [44]. By the third day of treatment, both total and LDL-C levels were significantly lower in the acute setting (p < 0.05) [44]. Despite the noticeable dose-dependent increases in bile acid and cholesterol synthesis, the study also found that cholestyramine therapy did not raise serum TGs in healthy people (transient hypertriglyceridemia was noted when treatment was initiated; however, this effect was not sustained). Serum TGs, glucose, and insulin levels increased (p < 0.05) in the acute context, but powerful farnesoid X receptor (FXR) agonistic bile acids (CDCA and DCA) temporarily decreased significantly [44].

Similar to colestipol, cholestyramine might have higher GI side effects and interactions with medications when compared to colesevelam. Additionally, cholestyramine can be used to treat pruritus related to partial biliary blockage [41]. The findings are summarized in Table 6.

**Table 6:** Role of bile acid sequestrants in the management of dyslipidemia LDL-C: low-density lipoprotein-cholesterol; A1C: glycated hemoglobin A1c; GI: gastrointestinal; T2DM: type 2 diabetes mellitus.

Medication, author, year, country	Study population	Mechanism of action	Benefits	Adverse effects
Colesevelam: Gavin et al., 2014, USA [42]	(≥65 years, N = 154) and (<65 years, N = 381)	Binding bile acids in GI tract lowers LDL-C by disrupting circulation and boosting bile production	Reduces LDL-C and A1C in both older and younger patients with hyperlipidemia and T2DM	Constipation (<65 years age = 10.3%, >65 years age = 13.4%), dyspepsia (8.8%, 6.7%), nausea (4.7%, 2.6%), accidental injury (3.4%, 4.6%), asthenia (4.1%, 2.1%), pharyngitis (3.6%, 1.0%), flu syndrome (3.4%, 2.1%), rhinitis (3.1%,3.6%), myalgia (2.6%, 0.5%)
Colestipol: Sando and Knight, 2015, USA [43]	N = 2278	Binding bile acids in GI tract lowers LDL-C by disrupting circulation and boosting bile production	Reduces total and LDL-C levels in patients with hypercholesterolemia	Mild GI effects. Edema, syncope, skin rashes have been reported [41]
Cholestyramine: Sjöberg et al., 2017, Sweden [44]	N = 9	Binding bile acids in GI tract lowers LDL-C by disrupting circulation and boosting bile production	Reduces total serum cholesterol and LDL-C levels. Treats pruritus related to partial biliary blockage	GI side effects. Can cause hyperchloremia acidosis [41]

Proprotein Convertase Subtilisin/Kexin Type 9 Inhibitors (PCSKi)

PCSK9 is an enzyme that binds to the LDL-Rs on hepatocytes, transferring them into endosomes and lysosomes where they are degraded. This causes more LDL to be present in the blood [45,46]. Therefore, therapies that target PCSK9 can prove useful in improving lipid profiles. As such, the FDA approved the use of alirocumab and evolocumab as PCSK9 inhibitors in 2015 [46].

Alirocumab: Alirocumab is a fully human IgG2 monoclonal antibody that binds to PCSK9, preventing it from interacting with LDL-Rs [45]. In a review article published in 2018, the authors describe the effectiveness of alirocumab via pooled results from five phase III trials. The patients were diabetic with dyslipidemia, which is unique in the sense that these patients are at higher risk for metabolic disorders and CVD due to traditional therapies not being able to achieve LDL, HDL, and Apo B target levels. In the ODYSSEY DM-INSULIN trial, alirocumab was able to significantly reduce LDL-C in type 1 patients and type 2 patients (type 1: 47.8% ± 6.5% versus placebo; type 2: 49.0% ± 2.7% versus placebo; both p < 0.0001). Apo-B and non-HDL were also reduced significantly (p < 0.0001). Alirocumab was associated with mild respiratory infections and injection-site reactions in only a minority of patients [45]. In the ODYSSEY DM-DYSLIPIDEMIA trial, results to date show that diabetic patients with mixed hyperlipidemia had a 33.3% reduction in non-HDL cholesterol for the group using alirocumab compared to fenofibrate (p < 0.0001) [46]. Evolocumab was associated with injection-site reactions and myalgia in some cases [46]. Ultimately, the authors analyzing these trials concluded that alirocumab is very effective in reducing cardiovascular events and lowering harmful lipid levels for patients with diabetic dyslipidemia.

Evolocumab: Evolocumab is a fully human IgG1 monoclonal antibody that also binds to and inhibits the function of PCSK9 [45]. This agent’s effectiveness in reducing LDL levels was studied in the FOURIER trial. This trial consisted of diabetic and non-diabetic patients with a median baseline level of 92 mg/dL. Evocolumab was shown to reduce LDL-C by 57% (95% CI: 56-58; p < 0.0001), Apo B by 48%, non-HDL cholesterol by 50% (95% CI: 49-51; p < 0.0001), and TGs by 16% (95% CI: 14-18, p < 0.0001) in diabetic patients [46]. This led to a significant 27% reduction in heart attack risk (p < 0.001), a 21% reduction in stroke risk (p = 0.01), and a 22% reduction in coronary revascularization risk (p < 0.001) [46]. Evolocumab was associated with injection-site reactions and myalgia in some cases [47]. Finally, the authors of this article highlighted how evolocumab did not raise hemoglobin A1C levels in diabetic patients during the FOURIER trial, a distinction from how statins raise these levels in other trials [46]. The findings are summarized in Table 7.

**Table 7:** Role of PCSK9 inhibitors in the management of dyslipidemia T1DM: type 1 diabetes mellitus; T2DM: type 2 diabetes mellitus; PCSK9: proprotein convertase subtilisin/kexin type 9; LDL-C: low-density lipoprotein-cholesterol; HDL-C: high-density lipoprotein-cholesterol; Apo-B: apolipoprotein B.

Medication, author, year, country	Study population	Mechanism of action	Benefits	Adverse effects
Alirocumab: Zhang et al., 2018, USA [46]	N = 517	Inhibits function of PCSK9 by working as an antibody	Reduction in LDL-C, Apo-B, and non-HDL-C serum levels. Reduction of negative cardiovascular events in patients with T1DM and T2DM	Injection-site reactions (3%) and mild respiratory infections (≥5%)
Evolocumab: Zhang et al., 2018, USA [46]	N = 27,564	Inhibits function of PCSK9 by working as an antibody	Reduction in LDL-C, Apo-B, and non-HDL-C serum levels. Reduction in heart attack, stroke, and coronary revascularization in all patients	Injection-site reactions (2.1%) and myalgia

Omega-3 Fatty Acids

Omega-3 fatty acids are polyunsaturated molecules that are ingested through various food sources. The consumption of fish provides eicosapentaenoic acid (EPA) and docosahexaenoic acid (DHA). Alpha-linolenic acid (ALA) is another omega-3 fatty acid that is found in vegetable sources such as flaxseeds and canola oil [43]. Omega-3 fatty acids are theorized to reduce TG synthesis, causing a reduction in VLDL-C production and leading to greater rates of VLDL-C metabolism. Antithrombotic and anti-inflammatory effects are also seen, boosting the health of arterial vessels [43]. Therefore, supplementation of omega-3 fatty acids is utilized in patients with severe hypertriglyceridemia or with a high risk of CVD. This is most often done in the form of a combined, ethyl ester formulation of both EPA and DHA or EPA alone. Although these effects sound promising, there have been mixed results as to whether omega-3 fatty acids reduce the risk of serious health problems that come with dyslipidemia.

EPA + DHA: Various formulations of EPA and DHA have been tested for the treatment of dyslipidemia. For example, a carboxylic acid combination of 0.550 g of EPA and 0.200 g of DHA in a 1 g capsule was tested in the STRENGTH trial [48]. Participants of this study received either 4 g/d of EPA+DHA or 4 g/d of corn oil along with any statins that patients may have used. However, the primary end point of either cardiovascular death, MI, stroke, coronary revascularization, or unstable angina was occurring at too high a rate. The study was halted early once 1384 out of the 13,078 patients achieved this endpoint. Seven hundred and eighty-five patients (12.0%) of the omega-3 group and 795 (12.2%) of the corn oil group hit the endpoint (HR, 0.99 [95% CI: 0.90-1.09]; p = 0.84) [48]. There were also more GI events in the omega-3 group (24.7% vs 14.7%) [48]. Ultimately, there was no significant statistical difference in the reduction of severe cardiovascular events between the two groups, and the drug was discontinued [48].

Other formulations are still in use, however, such as a drug formulation of 465 mg of EPA and 375 mg of DHA in a 1 g capsule. Although the aforementioned combination of 0.550 g of EPA and 0.200 g of DHA in a 1 g capsule was abandoned, different studies have shown the usefulness of EPA/DHA therapies. In the GISSI-Prevenzione trial, patients were given either 882 mg/day of an EPA and DHA combination, vitamin E 300 mg/day, both therapies, or none of the described therapies [43]. The group given the EPA/DHA therapy alone had a 10% reduction in the primary outcome, which was death or stroke, when compared in a two-way factorial analysis (p = 0.048) and a 15% reduction in a four-way analysis (p = 0.023) [43]. Thus, curating the optimal combination of EPA and DHA to significantly reduce complications is an endeavor that is always being tested in trials and studies.

Eicosapentaenoic acid: EPA, without the addition of DHA, has been another therapy utilized to reduce TG levels. The efficacy of such treatments was explored in the JELIS trial which involved patients who had a total cholesterol level of 6.5 mmol/L or higher [43]. Participants were given either 1800 mg/day of EPA along with statin therapy or just statin therapy alone. The EPA group had a 19% relative risk reduction in major cardiovascular events (p = 0.011) [43]. Therefore, EPA as a monotherapy can be another useful tool in controlling dyslipidemia. The findings are summarized in Table 8.

**Table 8:** Role of omega-3 fatty acids in the management of dyslipidemia EPA: eicosapentaenoic acid; DHA: docosahexaenoic acid; TG: triglyceride; VLDL-C: very low-density lipoprotein-cholesterol; GI: gastrointestinal.

Medication, author, year, country	Study population	Mechanism of action	Benefits	Adverse effects
EPA + DHA: Sando and Knight, 2015, USA [43]	N = 11,324	Inhibition of hepatic TG synthesis	Reduction in VLDL-C levels	Mild GI effects
EPA: Sando and Knight, 2015, USA [43]	N = 18,645	Inhibition of hepatic TG synthesis	Reduction in VLDL-C levels	Mild GI effects
EPA + DHA: Nicholls et al., 2020, USA [48]	N = 13,078	Inhibition of hepatic TG synthesis	Reduction in VLDL-C levels	6,532 people with EPA + DHA: diarrhea (11.9%), nausea (3.2%), dyspepsia (1.4%), abdominal discomfort (1.3%), diabetes onset (14.8%), syncope (0.5%), bleeding event (4.9%), major bleeding event (0.8%)

Niacin/Vitamin B3

Niacin is a lipid-lowering agent that was FDA-approved in 1997 for various indications; however, in 2016, the FDA withdrew approval for niacin use in combination with statins for high cholesterol. It treats dyslipidemia by inhibiting diacylglycerol acetyltransferase-2 (DGAT-2i), increasing acylation of fatty acids, reducing TG creation, and degrading additional ApoB, further lowering LDL levels [49]. It has been shown to reduce LDL-C levels by 5-25%, reduce TG levels by 20-25%, and increase HDL-C levels by 15-35% [50]. Side effects include flushing (seen in 90% of patients), hepatotoxicity, hyperglycemia, and diabetes risk [50]. A 211,567 community-based cohort study in Korea demonstrated that there was an association between high niacin intake and reduced risk of dyslipidemia (pooled, multivariable-adjusted HR: 0.71, 95% CI: 0.62-0.82) [49]. Additionally, spline regression demonstrated a dose-response linear relationship between dyslipidemia risk and niacin intake (where all p-values for nonlinearity >0.05) [49]. The findings are summarized in Table 9.

**Table 9:** Role of niacin/vitamin B3 in the management of dyslipidemia DGAT-2: diacylglycerol acetyltransferase-2; LDL: low-density lipoprotein-cholesterol; Apo-B: apolipoprotein B; TG: triglycerides; VLDL: very low-density lipoprotein-cholesterol.

Medication, author, year, country	Study population	Mechanism of action	Benefits	Adverse effects
Niacin/Vitamin B3: Kim and Park, 2022, Korea [49]	N = 211,567	Inhibiting DGAT-2, enhancing fatty acid acylation, reducing triglycerides, and degrading ApoB to lower LDL	Inhibiting lipolysis, lowering TG production in Liver, and decreasing VLDL levels	Flushing (seen in 90% of patients), hepatotoxicity, hyperglycemia, and diabetes risk

Metreleptin 

Metreleptin is a recombinant methionyl human leptin that is almost identical to the natural human leptin protein. However, it has an increased half-life compared to its natural counterpart due to the additional presence of a methionyl residue [51]. It is primarily utilized in the treatment of lipid deficiency, as well as lipodystrophy, a group of rare disorders with complications such as hypertriglyceridemia, dyslipidemia, and insulin-resistant diabetes [52]. A clinical study by Kinzer et al. demonstrated that those taking metreleptin for six months experienced a mean reduction in total cholesterol of -101.6 ± 95.6 mg/dL (p < 0.05), a mean reduction in TGs of −225.5 mg/dL (p < 0.05), and a mean reduction in LDL-C, measured by nuclear magnetic resonance, of −22.3 ± 12.8 mg/dL (p < 0.05) [53]. Therefore, metreleptin can potentially be used to significantly improve dyslipidemia. Furthermore, metreleptin is generally considered safe. Rodriguez et al. note concerns regarding lymphoma risk with metreleptin, but no definitive causal relationship has been proven. An uncommon event is the loss of drug efficacy. Other adverse effects, with a frequency of greater than 5%, include hypoglycemia, weight loss, and abdominal pain [51]. The findings are summarized in Table 10.

**Table 10:** Role of metreleptin in the management of dyslipidemia LDL-C: low-density lipoprotein-cholesterol.

Medication, author, year, country	Study population	Mechanism of action	Benefits	Adverse effects
Metreleptin: Kinzer et al., 2019, USA [53]	N = 17	Recombinant methionyl human leptin	Triglyceride reduction: −225.5, LDL-C reduction: −22.3 ± 12.8; total cholesterol reduction: −101.6 ± 95.6	Hypoglycemia, weight loss, abdominal pain, bloating, alopecia, confusion, anxiety, blurred vision, headache, nausea, and upper respiratory infection

Novel treatments

Bempedoic Acid

Bempedoic acid is a novel lipid-lowering medication to lower LDL-C levels in patients with HeFH and ASCVD [10]. It is a prodrug whose active form (bempedoyl coenzyme A) inhibits ATP Citrate Lyase, an enzyme involved in the cholesterol synthesis pathway by converting citrate into acetyl-CoA. Bempedoic acid elevates LDL-R synthesis and lowers cholesterol synthesis through ACLi, thereby improving serum LDL clearance. Because of this mechanism, it is particularly useful in controlling dyslipidemia in patients who are intolerant to statins or who need extra LDL-C reduction even after receiving maximally tolerated statin therapy. The primary side effects of bempedoic acid include hyperuricemia, nasopharyngitis, and urinary tract infections [10]. In terms of efficacy, the 52-week CLEAR Harmony experiment, a randomized controlled study with 2230 patients, showed that bempedoic acid considerably decreased LDL-C levels in contrast to placebo. The mean LDL-C level at week 12 was 19.2 mg per deciliter, which was a change of -16.5% from baseline (difference vs. placebo in change from baseline, -18.1 percentage points; 95% CI: -20.0 to -16.1; p < 0.001). Additionally, this trial demonstrated that the safety profile of bempedoic acid was basically comparable to that of placebo, despite a higher incidence of gout (1.2% vs. 0.3%) and adverse events that resulted in discontinuation (10.9% vs. 7.1%) in the bempedoic acid group [54]. The findings are summarized in Table 11.

**Table 11:** Role of bempedoic acid in the management of dyslipidemia ACLi: ATP citrate lyase inhibitor; LDL-C: low-density lipid-cholesterol; AE: adverse effect.

Medication, author, year, country	Study population	Mechanism of action	Benefits	Adverse effects
Bempedoic acid: Ray et al., 2019, USA [54]	N = 2,230	ACLi	Controlling dyslipidemia in patients intolerant to statins or who need extra LDL-C reduction even after receiving maximally tolerated statin therapy	Hyperuricemia, nasopharyngitis, and urinary tract infections (>4%), serious AEs (14.5%), and gout (1.2%)

Pemafibrate

Pemafibrate is a recently developed fibrate-class drug. It acts as a selective peroxisome proliferator-activated receptor α (PPARα) modulator (SPPARMα), activating specific PPARs to decrease TG levels via LPL. The purpose behind the curation of this drug was to create a safer fibrate, elucidating a more efficient response with fewer adverse effects [55,56]. Pemafibrate was explored in a recent double-blind, randomized trial, named the PROMINENT trial, which consisted of 10,497 patients. Compared to the placebo, pemafibrate caused more adverse renal effects (1463 patients vs. 1347 patients; HR, 1.12; 95% CI: 1.04 to 1.20; p = 0.004), more venous thromboembolism events (71 patients vs. 35 patients; HR, 2.05; 95% CI: 1.35 to 3.17; p < 0.001), and less hepatic events (155 patients and 200 patients, HR, 0.78; 95% CI: 0.63 to 0.96; p = 0.02). The researchers concluded that pemafibrate did not significantly reduce adverse cardiovascular events, but it effectively reduced molecules such as TGs and VLDL [56]. Phase 2 studies also show that pemafibrate significantly lowered TGs and increased HDL more than PPARMαs, such as fenofibrate, in dyslipidemia patients [57]. The findings are summarized in Table 12.

**Table 12:** Role of pemafibrate in the management of dyslipidemia PPARα: peroxisome proliferator-activated receptor alpha; SPPARMα: selective PPARα modulator; TG: triglycerides; VLDL-C: very low-density lipoprotein-cholesterol; ApoC3: apolipoprotein C-III.

Medication, author, year, country	Study population	Mechanism of action	Benefits	Adverse effects
Pemafibrate: Pradhan et al., 2022, multinational (24 countries) [56]	N = 10,497	Selective PPARα modulator (SPPARMα): Activates PPARα to reduce triglycerides via lipoprotein lipase stimulation	TG reduction: -26.2%; VLDL-C: -25.8%; remnant cholesterol: -25.6%; ApoC3: -27.6%	Higher adverse renal events: 1,463 vs. 1,347 patients; higher venous thromboembolism risk: 71 vs. 35 patients; lower hepatic events: 155 vs. 200 patients

PCSK9 siRNA

Inclisiran: Inclisiran is an siRNA that disrupts the messenger RNA for PCSK9, preventing PCSK9 from being created. It also conjugates N-acetylgalactosamine so that it can go directly to the liver, the primary site of PCSK9 production. This effectively increases efficacy and reduces the side effects associated with the drug [58]. The ORION-9, ORION-10, and ORION-11 phase III trials aimed to showcase the efficacy and safety of inclisiran as an injection-based treatment. Specifically, the ORION-9 trial focused on patients with HeFH. The study found that patients who received 300 mg of inclisiran on four different days over 510 days had an average LDL-C reduction of 47.9% compared to placebo (95% CI -53.5 to -42.73; p < 0.001) [59]. All three trials evaluated the safety of this drug. In total, mild cases of bronchitis (4.3%) and injection-site reactions (5%) were higher in the inclisiran group than in the placebo group. Both groups had equivalent occurrences of back pain, arthralgia, urinary tract infection, increased creatine phosphokinase (CPK) levels, and hypertension [59]. Overall, inclisiran has been shown to be a promising, safe non-statin therapy that patients with high LDL-C levels can consider.

Lerodalcibep: Lerodalcibep is a third-generation PCSK9i that has demonstrated consistent and significant LDL-C reduction in multiple dyslipidemia populations such as HeFH, ASCVD, and HoFH. In the LIBerate-HeFH trial by Raal et al., lerodalcibep reduced LDL-C by 58.61% (95% CI: -65.0% to -52.2%, p < 0.0001) at 24 weeks, with 68% of patients achieving LDL-C targets, and additional reductions in ApoB (-45.6%, p < 0.0001) and Lp(a) (-24.4%, p < 0.0001) [60]. Raal et al.’s follow-up study of LIBerate-HoFH in homozygous HF demonstrated modest reductions of LDL-C of -4.9% with lerodalcibep vs. -10.3% with evolocumab (95% CI: -0.2% to 11.1%), suggesting the limited efficacy of the drug in patients with minimal LDL-R function [61]. In contrast, a LIBerate-CVD trial by Kereiakes et al. demonstrated a 62% LDL-C reduction at 52 weeks (p > 0.0001) and >90% achieving <55 mg/dL LDL-C levels. Their study suggests long-term sustained efficacy in ASCVD patients [62]. Safety was favorable across all three studies, with mild injection-site reactions and no increase in serious adverse events. These studies position lerodalcibep as a promising therapy across a multitude of dyslipidemias. The findings are summarized in Table 13. 

**Table 13:** Role of PSCK9 inhibitors in the management of dyslipidemia PCSK9: proprotein convertase subtilisin/kexin type 9; LDL-C: low-density lipoprotein-cholesterol; siRNA: small interfering RNA; HeFH: heterozygous familial hypercholesterolemia; ASCVD: atherosclerotic cardiovascular disease; ApoB: apolipoprotein B; Lp(a): lipoprotein(a); UTI: urinary tract infection; ESC: European Society of Cardiology; HoFH: homozygous familial hypercholesterolemia; SAEs: serious adverse events.

Medication, author, year, country	Study population	Mechanism of action	Benefits	Adverse effects
Inclisiran: Albosta et al., 2023, USA [59]	N = 3,655	siRNA therapy targeting PCSK9 mRNA, preventing PCSK9 synthesis and increasing LDL receptor recycling	LDL-C reduction: -47.9% in ORION-9 (HeFH patients); -52.3% in ORION-10; -49.9% in ORION-11 (ASCVD patients)	Mild bronchitis (4.3%) and injection-site reactions (5%) occurred more in the inclisiran group vs. placebo; back pain, arthralgia, UTI, increased creatine phosphokinase, and hypertension were similar across groups
Lerodalcibep: Raal et al., 2023, multinational [60]	N = 478 in LIBerate-HeFH trial	Third-generation small recombination fusion protein inhibiting PCSK9	LDL-C reduction at 24 weeks: -58.61% ApoB reduction: -45.6%; Lp(a) reduction: -24.4%; 68% of patients achieved LDL-C <1.4 mmol/L	Mild injection-site reactions, similar to placebo; no significant safety concerns
Lerodalcibep: Raal et al., 2025, multinational [61]	N = 66 in LIBerate-HoFH trial	Third-generation small recombination fusion protein inhibiting PCSK9	LDL-C reduction at 24 weeks: -4.9% with lerodalcibep, -10.3% with evolocumab; no significant difference between treatments	Injection-site reactions in 2% with lerodalcibep vs. 24% with evolocumab; no treatment-related SAEs
Lerodalcibep: Kereiakes et al., 2024, multinational [62]	N = 922 in LIBerate-CVD trial	Third-generation small recombination fusion protein inhibiting PCSK9	LDL-C reduction at 52 weeks: -62.0%; >90% achieved ESC LDL-C target of <55 mg/dL	Mild injection-site reactions, no increase in serious adverse events (SAEs)

*Apolipoprotein C-III (apoC-III) siRNA* 

ApoC3 siRNA therapies effectively lower TG levels by reducing hepatic expression of ApoC3, which is a key regulator in TG metabolism by inhibiting LPL activity and liver clearance of TG-rich lipoproteins. Elevated ApoC3 levels have previously been associated with hypertriglyceridemia, increased CVD risk, and pancreatitis risk [63,64]. 

Plozasiran: A recent clinical study by Ballantyne et al. investigated a new siRNA interference agent targeting APOC3 called plozasiran in patients with mixed hyperlipidemia. Their plozasiran phase 2b trial demonstrated significant reductions in TG levels by 49.8% at 10 mg (95% CI: -59.0 to -40.6), 56% at 25 mg (95% CI: -65.1 to -46.8), 62.4% at 50 mg quarterly (95% CI: -71.5 to -53.2), and 44.2% at 50 mg half-annually ( 95% CI: -53.4 to -35.0) (p < 0.001 for all doses). Furthermore, plozasiran demonstrates significant reductions in APOC3 levels of 57.3% at 10 mg (95% CI: -66.6 to -48.1), 72.5% at 25 mg (95% CI: -81.7 to -63.3), and 78.5% at 50 mg (95% CI: -87.8 to -69.3) (p < 0.001 for all doses), with non-HDL cholesterol and Apo-B reductions of 16.7-24.2% (p < 0.001) and 10.3-19.1% (p < 0.001), respectively. Their trial demonstrated no elevations in liver enzymes or changes in platelet counts. However, worsening glycemic control was seen in 10% (placebo), 12% (10 mg), 7% (25 mg), 20% (50 mg), and 21% (50 mg half-yearly) of patients [63]. The SHASTA-2 phase 2b trial by Pall et al. is another trial that demonstrated plozasiran to have significant effects in reducing TG levels by 74% (p < 0.0001) and APOC3 levels (p > 0.0001) by 78% in patients with severe triglyceridemia. Their study showed the long-term sustained effects of lowering TGs, with more than 90% of plozasiran-treated patients achieving TG levels below 500 mg/dL by 24 weeks and 78% by 48 weeks (p > 0.0001). No significant adverse events were reported with no patients discontinuing the drug or deaths [64]. These studies conclude that plozasiran is a promising and robust candidate in not only acute but sustained reduction of TG levels and improvement in atherogenic lipid profiles. These findings support further advancements of plozasiran into phase 3 trials to determine safety and efficacy. The findings are summarized in Table 14.

**Table 14:** Role of ApoC3 siRNA in the management of dyslipidemia TG: triglycerides; APOC3: apolipoprotein C-III; SiRNA: small interfering RNA; mRNA: messenger RNA; non-HDL-C: non-high-density lipoprotein-cholesterol; ApoB: apolipoprotein B; ALT: alanine aminotransferase; AST: aspartate aminotransferase; SAE: serious adverse event.

Medication, author, year, country	Study population	Mechanism of action	Benefits	Adverse effects
Plozasiran: Ballantyne et al., 2024, USA, Canada, Australia, Europe, and New Zealand [63]	N = 353	SiRNA interference agent targeting APOC3 mRNA	TG reduction: -49.8% to -62.4%; APOC3 reduction: -57.3% to -78.5%; non-HDL-C: -16.7%, -17.5%, -24.2%; ApoB reduction: -10.3%, -13.0%, -19.1%	Worsening glycemic control; no significant changes in platelet count, ALT/AST levels; 4 deaths (not treatment-related)
Plozasiran: Pall et al., 2024, USA, Canada, Australia, Europe, New Zealand [64]	N = 229	SiRNA interference agent targeting APOC3 mRNA	TG reduction: -74%; APOC3 reduction: -78%; sustained reduction at 48 weeks: TG -58%, APOC3 -48%; TG <500 mg/dL achieved in >90% of patients by 24 weeks, 78% sustained at 48 weeks; durable reductions in remnant cholesterol, non-HDL-C, and ApoB through 48 weeks	No significant increase in adverse events; all serious adverse events (SAEs) were mild to moderate (grade 1-3); no SAEs led to discontinuation or death

*Cholesterol Ester Transfer Protein Inhibitors (CETPis)* 

Cholesteryl ester transfer protein (CETP) transfers cholesteryl esters from HDL-C particles to LDL-C, VLDL-C, and other Apo-B-containing lipoproteins in exchange for TGs. These proteins play an essential role in lipid metabolism and are associated with a higher risk of atherosclerosis and CVD by redistributing the concentration of HDL-C to LDL-C and VLDL-C. CETP inhibitors, such as CKD-519, obicetrapib, evacetrapib, and torcetrapib, block this transfer and as a result, will increase HDL-C and decrease LDL-C serum levels. These drugs were developed to reduce the CVD risk beyond what can be achieved from conventional statin therapy [65]. 

CKD-519: A clinical study by Kim et al. explored a potent CETPi called CKD-519. Their studies showed 63-83% inhibition of CETP activity and a 25-48% increase in HDL with doses varying from 25 to 400 mg (p < 0.05). Within the study, there were 13 adverse events, which varied from headache, hypertriglyceridemia, diarrhea, nausea, dizziness, periodontal disease, allergic rhinitis, and elevated CPK. Out of these events, increased CPK and hypertriglyceridemia were considered possibly related to CKD-519 [65]. Although the study mentioned that single doses of CKD-519 400 mg were well tolerated by healthy individuals, the array of adverse events necessitates further research into the safety and efficacy of this medication. 

Obicetrapib: Another promising CETPi candidate is obicetrapib. Ballantyne et al.’s phase 2 trial, which combined 10 mg obicetrapib with 10 mg ezetimibe, showed a significant reduction in LDL-C through combination therapy, monotherapy, and placebo groups, 63.4%, 43.5%, and 6.35%, respectively (p < 0.0001) [66]. Obicetrapib in combination with ezetimibe significantly lowered atherogenic lipid parameters and is an encouraging, upcoming therapy, as there were no safety issues identified in the study. 

Evacetrapib and torcetrapib: A recent clinical study by Furtado et al. analyzed whether two other CETPis, evacetrapib and torcetrapib, would also lead to beneficial lipid profile shifts. Their clinical trials demonstrated that evacetrapib and torcetrapib increase HDL-C by 125% and 29%, respectively. Their research also demonstrated that evacetrapib and torcetrapib resulted in an increase in apoA1 lipoprotein by 99% and 50%, respectively (p < 0.001). However, their study also found a disproportionately enhanced subspecies of apoC3 lipoprotein, with the proportion of total HDL containing apoC3 increasing by 16.5% for patients on torcetrapib and 18.4% for patients on evacetrapib (p < 0.05). This HDL-C apoC3 subtype has been previously associated with higher CVD risk [67].

Anacetrapib: Sammons et al. conducted a comprehensive analysis as part of the HPS3/TIMI55-REVEAL trial on another CETPi candidate called anacetrapib on atherosclerotic vascular disease (AVD) and lipid metabolism. Their trial consisted of 30,449 participants with AVD randomized to either 100 mg of anacetrapib daily or placebo. Their in-trial treatment period of median 4.1 years discovered that anacetrapib reduced the incidence of first major coronary events by 9% (rate ratio 0.91; 95% CI 0.85-0.97; p = 0.004), and their post-trial follow-up period (median 2.3 years) showed a further reduction of major coronary events by 20% (rate ratio 0.80; 95% CI 0.71-0.90; p < 0.001). Furthermore, anacetrapib increased mean HDL-C levels by 43 mg/dL and decreased mean non-HDL-C cholesterol levels by 17 mg/dL at the midpoint of the in-trial treatment period. Additionally, these lipid modifications persisted into the post-treatment period, with mean HDL-C levels remaining 44 mg/dL higher and mean non-HDL-C levels 17 mg/dL lower. This study reported a minor increase in systolic blood pressure of 0.7 mmHg and an increase of 0.3 mmHg in diastolic blood pressure, but not correlated to an increase in hypertensive adverse events and no significant adverse effects on non-vascular mortality [68]. 

The findings of the studies on the various CETPis such as CKD-519, obicetrapib, evacetrapib, torcetrapib, and anacetrapib have shown promising improvements in lipid parameters, such as increasing HDL-C and lowering LDL-C levels. However, there are some candidates, such as evacetrapib and torcetrapib that displayed off-target effects that may limit the clinical benefits. This complexity necessitates more research to determine the safety and efficacy of treating dyslipidemia. The findings are summarized in Table 15.

**Table 15:** Role of CETP inhibitors therapy in the management of dyslipidemia CETP: cholesteryl ester transfer protein; HDL-C: high-density lipoprotein-cholesterol; LDL-C: low-density lipoprotein-cholesterol; CVD: cardiovascular disease.

Medication, author, year, country	Study population	Mechanism of action	Benefits	Adverse effects
CKD-519: Kim et al., 2016, Korea [65]	N = 40	Selectively inhibits CETP activity	63-83% inhibition of CETP activity and a 25-48% increase in HDL with doses varying from 25 to 400 mg	Headache (two cases), hypertriglyceridemia (one case), diarrhea (one case), nausea (one case), dizziness (one case), increased creatine phosphokinase levels (two cases), dyspepsia (one case), periodontal disease (one case), allergic rhinitis (one case), dyspnea (one case), and conjunctivitis (one case)
Obiceptrapib: Ballantyne et al., 2023, USA [66]	N = 97	Inhibits CETP activity, used as monotherapy or in conjunction with ezetimibe for LDL-C reduction	LDL-C reduction of 63.4% in combination therapy, 43.5% in monotherapy, 100% achieved LDL-C <100 mg/dL	Well tolerated, no safety issue reported
Evacetrapib and torcetrapib: Furtado et al., 2022, USA [67]	ACCENTUATE trial (N = 126); Illuminate trial (N = 80)	Inhibitors CETP-mediated cholesteryl ester transfer, increasing HDL-C and ApoA1	HDL-C increased by 125% with evacetrapib and 29% with torcetrapib	Increased levels of dysfunctional HDL subspecies associated with higher CVD risk
Anacetrapib: Sammons et al., 2022, UK [68]	N = 30,449	Inhibitors CETP-mediated cholesteryl ester transfer, increasing HDL-C and reducing LDL-C	9% reduction in major coronary events during a 4.1-year treatment. 20% reduction during a 2.3-year post-trial follow-up. Sustained lipid benefits: HDL-C +43 mg/dL, non-HDL-C −17 mg/dL at midpoint	Minimal adverse effects: Slight increase in systolic BP (0.7 mmHg). No significant non-vascular mortality or major adverse events

*Antisense Oligonucleotide Therapies* 

Antisense oligonucleotides (ASOs) are synthetic single-stranded oligonucleotides that bind to complementary mRNA, causing RNase H1-mediated cleavage of the mRNA. This ultimately prevents the translation of lipoproteins [69]. 

AKCEA-APO(a)-LRx: A clinical study by Langsted and Nordestgaard focused on phase 2b development of an ASO that specifically targeted lipoprotein(a) (Lp(a)) called AKCEA-APO(a)-LRx. Lp(a) was specifically chosen as elevated levels were linked to an increased risk of MI and aortic valve disease. Their study has shown that AKCEA-APO(a)-LRx reduced Lp(a) levels by 80% with a 20 mg weekly dose, 56% with a 40 mg biweekly dose, and 72% with a 60 mg monthly dose. Even though this drug candidate demonstrated significant lipid-lowering effects, the cardiovascular outcomes are still in the process of being evaluated [69]. 

Olezarsan: The Bridge-TIMI 73a trial by Bergmark et al. demonstrated a novel drug called olezarsan, an ASO that targets APOC3 mRNA, as an effective candidate for reducing TG and APOC3 in patients with moderate to severe hypertriglyceridemia. Their randomized placebo-controlled phase 2b study demonstrated TG levels reduction by 49.3% (95% CI: 39.5 to 59.0) and 53.1% (95% CI: 43.4 to 62.9) at 50 mg and 80 mg, respectively, at six months (p < 0.001 for both). ApoC3 was reduced by 64.2% and 73.2% at 50 mg and 80 mg, respectively (p < 0.001), while non-HDL cholesterol and Apo-B levels were reduced by 18.2% and 9.9% (50 mg) and 25.4% and 9.8% (80 mg), respectively (p < 0.001). However, their study found significant reductions in platelet count below 140,000/uL occurring in 17% (50 mg) and 18% (80 mg) of patients (p = 0.03 and 0.04, respectively). The authors noted no severe cases of thrombocytopenia below 75,000/uL. Additionally, significant transaminitis occurred in 47% (50 mg) and 37% (80 mg) (p > 0.001 for both) [70]. These findings position olezarsen as a candidate in effectively lowering TG and atherogenic lipid profiles; however, pending phase 3 trials will be required to further assess the adverse effects and long-term cardiovascular outcomes. The findings are summarized in Table 16.

**Table 16:** Role of ASO therapies in the management of dyslipidemia ASOs: antisense oligonucleotides; LDL-C: low-density lipoprotein-cholesterol; HoFH: homozygous familial hypercholesterolemia; ANGPTL3: angiopoietin-like protein 3; PCSK9: proprotein convertase subtilisin/kexin type; LFTs: liver function tests.

Medication, author, year, country	Study population	Mechanism of action	Benefits	Adverse effects
AKCEA-APO(a)-LRx: Langsted and Nordestgaard, 2019, Denmark [69]	N = 286	Utilizes ligand-conjugated (LICA) technology for target	56%-80% reduction in Lp(a) with AKCEA-APO(a)-LRx	Injection-site reaction in ~26% of patients, with elevated LFTs. Hepatic steatosis and bleeding risk were not observed.
Olezarsen: Bergmark et al., 2024, USA and Canada [70]	N = 154	ASO targeting APOC3 mRNA	TG reduction: -49.3% (50 mg), -53.1% (80 mg); APOC3 reduction: -64.2%, -73.2%; non-HDL-C reduction: -18.2%, -25.4%; ApoB reduction: -9.9%, -9.8%	ALT elevations: 47% (50 mg), 37% (80 mg). Platelet count <140,000/μL: 17% (50 mg), 18% (80 mg). No severe thrombocytopenia

*Angiopoietin-Like Protein 3 Inhibitors (ANGPTL3i)* 

ANGPTL3 (angiopoietin-like protein 3) is a glycoprotein that is secreted predominantly by the liver. ANGPTL3’s key role is inhibiting LPL and endothelial lipase (EL), and as a result, increasing circulating levels of TGs, LDL-C, and other lipoproteins. The rise in TG-rich lipoproteins is a causative factor in developing dyslipidemia [6]. A population study by Stitziel et al. demonstrated a 34% reduced risk of CAD found among carriers of an ANGPTL3 loss-of-function (LOF) mutation compared to noncarriers (OR: 0.66; 95% CI: 0.44-0.98; p = 0.04). Heterozygous carriers of the LOF mutation demonstrated a 17% reduction in circulating TGs and a 12% reduction in LDL-C. These findings indicate that ANGPTL3 might influence the progression of CAD [71].

Evinacumab: Given the regulatory role of ANGPTL3, there is ongoing research to develop inhibitors of ANGPTL3 to address dyslipidemia and reduce CVD risk. Evinacumab is a monoclonal antibody that targets and inhibits ANGPTL3 to treat familial hypercholesterolemia. Phase 3 clinical trials of evinacumab by Sosnowska et al. showed a 47.1% reduction in LDL-C (95% CI: -65.0, -33.1; p < 0.001) with no major adverse effects reported during the trials. In patients with hypertriglyceridemia, evinacumab was able to reduce TG levels by 88.2% (p < 0.0003) [11]. Similarly, a review of phase 3 clinical trials (N = 65) by Kosmas et al. highlighted the efficacy of evinacumab in patients with HoFH and who had failed PCSK9 inhibitors, with an LDL-C reduction of 47.1% by week 24 compared to the placebo group of 1.9% (p < 0.001) [72].

E3 peptide vaccine: Fukami et al. explored a novel long-term solution to dyslipidemia and atherosclerosis by developing a peptide-based vaccine with epitopes targeting ANGPTL3. This study is within the preclinical phase and was performed on mice. The E3 peptide vaccine shows promising results, with the reduction of serum TG (p < 0.05), LDL-C (p < 0.01), and sd-LDL-C (p < 0.05), in obese mice with induced dyslipidemia. In another model of mice with several familial hypercholesterolemia (ApoE-deficient mice fed a high-cholesterol diet), there was a significant decrease in non-fasting TG levels by week-6 post-E3 immunization, as well as a substantial reduction in fasting LDL-C and VLDL-C levels (p < 0.01 for all). Notably, the E3 vaccine did not induce cytotoxic autoimmune responses and improved liver pathology by decreasing IL-6 and TNF-α markers (p < 0.05). These factors deem E3 as a promising, cost-effective long-term strategy for high-risk patients with resistant dyslipidemia [73]. These studies establish ANGPTL3 inhibitors as robust candidates for treating resistant hypercholesterolemia, especially in patients who do not adequately respond to statins, PCSK9 inhibitors, or other therapies. The findings are summarized in Table 17.

**Table 17:** Role of ANGPTL3 inhibitor therapies in the management of dyslipidemia LDL-C: low-density lipoprotein-cholesterol; sd-LDL-C: small dense low-density lipoprotein-cholesterol; HDL-C: high-density lipoprotein-cholesterol; TG: triglycerides; ANGPTL3: angiopoietin-like protein 3; HoFH: homozygous familial hypercholesterolemia; ApoB: apolipoprotein B; LFTs: liver function tests; CVD: cardiovascular diseases.

Medication, author, year, country	Study population	Mechanism of action	Benefits	Adverse effects
ANGPTL3 mutation study: Stitizel et al., 2017, USA, UK, Germany, Pakistan [71]	N = 180,180	Loss-of-function ANGPTL3 mutation	34% reduced risk of CVDs, 17% reduction in TG, and 12% reduction of LDL-C in ANGPTL3 LOF mutation	HDL metabolism is not altered, long-term effects of ANGPTL3 inhibition remains unknown
Evinacumab: Sosnowska et al., 2022, Poland [11]	N = 65	Binds and inhibits ANGPTL3, reducing LDL-C and TG	LDL-C reduced by 49%; triglycerides reduced by 55% in HoFH patients after 24 weeks of treatment.	Upper respiratory infections (6.5%), mild injection-site reactions (11%)
Evinacumab: Kosmas et al., 2022, USA/Greece [72]	N = 65	Binds and inhibits ANGPTL3, reducing LDL-C and TG	LDL-C reduction of 47.1% in HoFH patients	Mild injection-site reaction, no significant LFT elevation or cardiovascular adverse events
E3 peptide vaccine: Fukami et al., 2021, Japan [73]	N = 7-12	E3 peptide vaccine targeting the ANGPTL3 E3 epitope by producing antibodies to reduce activity	Reductions in non-fasting TG, LDL-C, and sd-LDL-C levels in ob/ob mice; decreased fasting TG, LDL-C, and VLDL-C in ApoE-deficient mice; improved hepatic lipid accumulation and reduced inflammatory markers (IL-6, TNF-α)	No cytotoxic response or systemic side effects observed in animal studies

SC401

A recent study analyzed the efficacy and safety of a novel formulation of docosahexaenoic acid ethyl esters (DHA-EEs) and eicosapentaenoic acid ethyl esters (EPA-EEs). This drug has 1530 mg of EPA-EEs and DHA-EEs (SC401), along with utilizing advanced lipid technologies to increase bioavailability [74]. The trial had 23 participants, consisting mostly of men, who were given an SC401 and a different formulation (3600 mg ω-3, primarily EPA-EEs and DHA-EEs) treatment sequence. Drug-related events for SC401 were identified as one case of diarrhea and one case of flatulence, while the alternative treatment had one case of abdominal pain and one case of eructation [74]. A 95% CI was utilized in this trial. Ultimately, the SC401 treatment was found to have a 197% to 295% higher peak concentration and area under the curve value than the alternative. The time it took to reach peak drug concentration was also shorter for SC401 at around 6 hours compared to 10 hours. These findings mean that SC401 was able to achieve a higher bioavailability of DHA and EPA in the body at a lower dose when compared to a different, known omega-3 fatty acid combination [74]. Long-term testing of SC401 for effects on TG levels and safety profiles needs to be further conducted. The findings are summarized in Table 18. 

**Table 18:** Role of SC401 in the management of dyslipidemia DHA-EEs: docosahexaenoic acid ethyl esters; EPA-EEs: eicosapentaenoic acid ethyl esters.

Medication, author, year, country	Study population	Mechanism of action	Benefits	Adverse effects
SC401: Lopez-Toledano et al., 2017, USA [74]	N = 23	ω-3 Acid ethyl ester (DHA-EEs and EPA-EEs) formulation increases bioavailability of DHA-EEs and EPA-EEs	SC401 had a 197% to 295% higher peak concentration; the alternative formulation; faster peak concentration time (6 hours vs. 10 hours), indicating enhanced absorption	Diarrhea, flatulence

Discontinued medications 

Mipomersen 

Mipomersen is an ASO that targets Apo-B, which reduces levels of molecules such as Lp(a) in circulation. A clinical phase 3 meta-analysis of mipomersen by Langsted and Nordestgaard demonstrated a 31% decrease of Lp(a) in 45 patients with HoFH, a 33% reduction in Lp(a) in 58 patients with moderately elevated LDL, a 24% reduction of Lp(a) in 158 patients at high risk of coronary heart disease, and a 21% decrease of Lp(a) in 124 patients with HeFH. The most common adverse event noted was injection-site reactions, seen in >90% of patients, and transaminitis. The authors expressed their concern about compromising liver function and hepatic steatosis [69]. Another clinical trial review study done by Yamamoto et al. highlights the efficacy of mipomersen, an ASO, in treating statin-resistant HoFH. Mipomersen targets the apolipoprotein B-100 (ApoB) mRNA and was among the first FDA-approved medications for lipid management due to its effect of reducing LDL-C and ApoB lipoproteins. The authors’ review of the mipomersen sodium injection study demonstrates that 200 mg/week over 26 weeks of mipomersen reduced LDL-C by -24.7% compared to placebo, -3.3%, in patients with HoFH. However, the authors note that adverse events of the drug included injection-site reactions, flu-like symptoms, increased transaminases, and steatosis. There was a 55% dropout rate of enrolled patients due to these adverse events [75]. Ultimately, the negative outcomes outweighed the benefits, and mipomersen has been removed from the market by the European Medicines Agency and the FDA [76]. 

AZD8233 

More recently, a phase 2B clinical trial by Hofherr et al. explored a novel PCSK9-targeted ASO, AZD8233. In 119 participants, AZD8233 reduced PCSK9 by 93% (95% CI: -95, -91; p < 0.001) and LDL-C by 79% with a 90 mg dose after 12 weeks (95% CI: -83, -74; p < 0.001). This drug was dosed less frequently and was reportedly well-tolerated with no significant adverse effects reported [77]. Nevertheless, despite these findings, AZD8233 was eventually abandoned because the results, particularly in terms of efficacy, were not satisfactory enough to warrant further development [78]. 

Vupanorsen 

Vupanorsen was an N-acetyl galactosamine-conjugated ASO targeting ANGPTL3 mRNA. Graham et al. evaluated vupanorsen in a phase 1 trial, which demonstrated a reduction of TG by 63.1% (p = 0.01) and ANGPTL3 protein levels by 84.5% (p = 0.001). Similarly, their study also observed a reduction in LDL-C by 32.9% (p < 0.001). This trial reported no serious adverse events, with only mild headaches and dizziness noted in both placebo and treatment groups [79]. Similarly, a randomized, double-blinded, placebo-controlled phase 2 study by Gaudet et al. demonstrated that patients receiving 80 mg of vupanorsen every four weeks experienced significant lipid-lowering effects, particularly reductions in TG by 53% (95% CI: -63%, -43%; p < 0.0001) and ANGPTL3 protein by 50% (95% CI: -68%, -50%; p < 0.0001). Vupanorsen also reduced apolipoprotein C-III by 58% (p < 0.0001) and remnant cholesterol by 38% (p < 0.0001) while showing minor injection-site reactions and no significant changes in blood sugar. Adversely, hepatic fat fraction was increased by 4% (p = 0.0230) [80]. After clinical trials, vupanorsen was discontinued in 2022 due to a significant increase in liver transaminase [81]. 

Bococizumab 

Bococizumab was a humanized monoclonal antibody that inhibited PCSK9 from decreasing LDL levels [82]. Researchers testing this drug conducted two trials in parallel (SPIRE-1 and SPIRE-2) where 27,438 patients randomly received either 150 mg of bococizumab subcutaneously every two weeks or a placebo drug. Results indicated that baseline levels of LDL levels were reduced by 56% in the bococizumab group (p < 0.001) and a median reduction of 64.2% (p < 0.001) [82]. Regarding cardiovascular events, the longer, higher-risk trial showcased that bococizumab might be of benefit compared to the placebo (new source). For the two trials combined, the HR for the end point was 0.88 (95% CI: 0.76 to 1.02; p = 0.08) [82]. However, these trials were never able to finish, as bococizumab was discontinued due to the high amount of anti-drug antibodies that were formed in the patients [82]. Additionally, the range of responses in LDL cholesterol changes was too high, further consolidating the decision to stop testing of this drug [83]. The findings are summarized in Table 19. 

**Table 19:** Discontinued medications mRNA: messenger ribonucleic acid; ApoB: apolipoprotein B; Apo(a): apolipoprotein A; ANGPTL3: angiopoietin-like protein 3; Lp(a): lipoprotein(a); HoFH: homozygous familial hypercholesterolemia; GaINAc: N-acetylgalactosamine; ASO: antisense oligonucleotide; PCSK9: proprotein convertase subtilisin/kexin type 9; LDL-C: low-density lipoprotein-cholesterol.

Medication, author, year, country	Study population	Mechanism of action	Benefits	Adverse effects
Mipomersen: Langsted and Nordestgaard, 2019, Denmark [69]	N = 385	Hybridize with mRNA to induce RNAase H-mediated cleavage, reducing ApoB, Apo(a), and ANGPTL3 levels	A 31% reduction Lp(a) in patients with HoFH, a 33% reduction in Lp(a) in patients with moderately elevated LDL, a 24% reduction of Lp(a) in patients at high risk of coronary heart disease, and a 21% decrease of Lp(a) in patients with HeFH	Injection-site reactions in >90% of the patients, flu-like symptoms, increased transaminases, and steatosis
Mipomersen: Yamamoto et al., 2016, Japan [75]	N = 382	Hybridize with mRNA to induce RNAase H-mediated cleavage, reducing ApoB, Apo(a), and ANGPTL3 levels	LDL-C reductions of 24.7% with mipomersen	Injection-site reactions, flu-like symptoms, increased transaminases, and steatosis, 55% dropout rate
AZD8233: Hofherr et al., 2022, USA/Sweden [77]	N = 119	GalNAc-conjugated ASO targets of PCSK9 mRNA	AZD8233 reduced PCSK9 by 93% and LDL-C by 79%	Mild injection-site reaction, no significant LFT elevation or cardiovascular adverse events
Vupanorsen: Graham et al., 2017, USA [79]	N = 44	N-acetyl galactosamine-conjugated antisense oligonucleotide targeting ANGPTL3 mRNA	Reductions in triglycerides (63.1%), ANGPTL3 protein (84.5%), LDL-C (32.9%), and non-HDL cholesterol (36.6%)	Headache and dizziness; no serious adverse events
Vupanorsen: Gaudet et al., 2020, USA [80]	N = 105	N-acetyl galactosamine-conjugated antisense oligonucleotide targeting ANGPTL3 mRNA	Reductions in triglycerides (53%), ANGPTL3 protein (59%), apolipoprotein C-III (58%), and remnant cholesterol (38%)	Injection-site pruritus and erythema; no serious adverse events
Bococizumab: Ridker et al., 2017, multinational [82]	N = 27,438	PCSK9 monoclonal humanized antibody	Reduction in LDL-C levels (56%)	Injection-site reactions; formation of anti-drug antibodies

## Conclusions

Dyslipidemia treatments are an ever-evolving subject of medicine. Strides have been made in curating novel and safe pharmacological agents to target LDL-C, ApoB, and other specific molecules contributing to the disease. One of the primary concerns with traditional agents such as statins involves the risk of rhabdomyolysis, SAMS, and drug intolerance. Specifically, the significant residual risk for CVD or the intolerance of statins in certain patients is addressed by researchers. 

Emerging treatments, such as lerodalcibep (PSCK9i), plozasiran (APOC3 siRNA), pemafibrate (SPPARMα), and inclisiran (PCSK9i), show promising results in reducing LDL-C and TG levels while minimizing the side effects and maintaining or even increasing the levels of efficacy in combination therapy. Lerodalcibep is a promising dyslipidemia therapy candidate with versatility in managing patients with HoFH, HeFH, and ASCVD. Early trials of plozasiran have shown a notable reduction in TG levels and favorable atherogenic lipid profiles with minimal adverse events. Pemafibrate was designed as a safer fibrate to decrease TG levels and raise HDL-C effectively. Inclisiran provides a significant and sustained reduction of LDL-C with a lower incidence of adverse events. 

These could be implemented as a combination therapy or replace traditional therapies. Certain drugs discussed in this article are already available for providers to prescribe to their patients. Although findings from clinical trials may differ in practice due to patient heterogeneity, the variety of medications discussed carries the potential to personalize lipid-lowering therapies to individual patient profiles. Clinical trials also predominantly emphasize the safety profiles of these advances. The trajectory of future research is to optimize therapeutic dosage and cost-effectiveness. Therefore, these promising new medications are on track to complement traditional dyslipidemia therapies, allowing for more treatment options that are tailored to the patient. 
